# Acceptability, feasibility, and program outcomes of an equity-focused, adapted community-based healthy lifestyle program for children, young people, and their families in Perth, Western Australia: an implementation hybrid research protocol

**DOI:** 10.3389/frhs.2025.1604809

**Published:** 2025-07-17

**Authors:** Stephanie Smith, Stephen Paull, Katie M. Iwanowski, Tania Harris, Joanna C. Moullin, Monica Jane, Jordan Bill, Deborah A. Kerr, Christina M. Pollard, Glenn Pearson, Melanie Robinson, Bonnie Furzer, Natasha Bear, Ben Jackson, Robyn Mildon, Nick Sevdalis, Richard Norman, Jacqueline A. Curran, Cameron C. Grant, Sarah C. Armstrong, Yvonne C. Anderson

**Affiliations:** ^1^ Curtin Medical School, Faculty of Health Sciences, Curtin University, Bentley, WA, Australia; ^2^ Child and Adolescent Community Health, Child and Adolescent Health Service, Perth, WA, Australia; ^3^ The Kids Research Institute Australia, Perth Children’s Hospital, Nedlands, WA, Australia; ^4^ Health Consumers’ Council, Perth, WA, Australia; ^5^ School of Population Health, Faculty of Health Sciences, Curtin University, Bentley, WA, Australia; ^6^ School of Human Sciences (Exercise and Sports Science), Faculty of Science, The University of Western Australia, Nedlands, WA, Australia; ^7^ Thriving in Motion, Perth, WA, Australia; ^8^ Institute for Health Research, Notre Dame University, Perth, WA, Australia; ^9^ Centre for Evidence and Implementation, Melbourne, VIC, Australia; ^10^ Centre for Behavioural and Implementation Science Interventions, National University of Singapore, Singapore, Singapore; ^11^ Department of Endocrinology & Diabetes, Perth Children’s Hospital, Perth, WA, Australia; ^12^ Department of Paediatrics: Child and Youth Health, Faculty of Medical and Health Sciences, University of Auckland, Grafton, Auckland, New Zealand; ^13^ Department of Pediatrics, Duke University School of Medicine, Durham, NC, United States; ^14^ Duke Center for Childhood Obesity Research, Durham, NC, United States

**Keywords:** community-based participatory research, healthy lifestyle, obesity, implementation science, health equity, program evaluation, feasibility studies

## Abstract

**Background:**

International guidelines recommend multidisciplinary intervention programs for addressing childhood obesity. In Western Australia, community-based healthy lifestyle programs for children and young people are insufficient for demand, especially for those over-represented in obesity statistics relating to prevalence. This protocol outlines the implementation and evaluation of an adapted, evidenced, community-based program in Perth, Western Australia.

**Methods:**

This study follows a multiple-methods hybrid type II design, testing acceptability and feasibility of program scale-out and program participant outcomes. *Study* (*1*) *Develop the adapted healthy lifestyle program with key partners and Aboriginal and Torres Strait Islander advisors for scale-out.* Apply implementation strategies for program realisation. Identify critical elements and outcomes to demonstrate program success with key partners (∼30 workshop participants and ∼80 qualitative proforma respondents). Identify cultural and place-based considerations for program acceptability with Aboriginal and Torres Strait Islander Elders/advisors (∼30 workshop participants). *Study* (2) *Assess acceptability, appropriateness, feasibility, and local clinical outcomes of adapted healthy lifestyle program pilot.* Evaluate the program with children and young people aged 4–16 years with obesity or overweight and weight-related comorbidities, seeking healthy lifestyle change. The program will include weight-related assessments at baseline, 6, and 12 months with weekly sessions for 6 months (estimated *n* = 245 over 22 months, accounting for 30% drop-out). Explore program experience via focus groups with children, young people, and caregivers: ∼8–12 weeks post commencement (∼50 program participants and caregivers), ∼6 months post commencement (∼50 program completers and caregivers), and evaluation survey (e.g., declined/dropped out/completed). Engage with key partners to determine program feasibility for scale-up (∼30 workshop participants and ∼80 qualitative proforma respondents). Mixed model regression will be used to assess within-subject change in outcomes over time. Child health utility instruments will be used for cost-utility analysis. *Study* (3) *Assess program scalability post-pilot.* If determined feasible and acceptable, the program will be packaged to assist practitioners and policymakers with scale-up via exploration of currently available packages and key staff interviews. The overarching Replicating Effective Programs framework outlines the implementation stages, and the tools and strategies being applied are presented. Qualitative data will be analysed using Framework Analysis, incorporating the Consolidated Framework for Implementation Research and the Expert Recommendations for Implementing Change.

**Discussion:**

Implementation outcomes will be determined by evaluating acceptability, appropriateness, and feasibility of scale for this healthy lifestyle program. Utilising implementation science, partnership-building, and place-based and cultural considerations, this research will inform potential scale-up of equity-focused healthy lifestyle programs.

## 1 Introduction

“This [excess weight] is such a massive issue for our mob, all over Western Australia and beyond. We’ve got to do better” (Aboriginal Leader, early program development)

In the past 40 years, the number of school-age children and adolescents living with obesity globally has increased more than eightfold (1). By 2035, it is estimated that 39% of children and young people aged 5–19 years globally will be living with overweight or obesity (2). In Australia, the prevalence rate is estimated to be 46% (2). In addition, the inequities caused by the inter-generational impact of colonisation have resulted in Aboriginal and Torres Strait Islander children and those experiencing socioeconomic disadvantage being over-represented in these estimates (3). Children above a healthy weight have an increased risk of numerous weight-related comorbidities, including obstructive sleep apnoea, hypertension, type 2 diabetes (4), and reduced quality of life (5). In Western Australia (WA) in 2019, there was an 18-fold higher incidence of type 2 diabetes in Aboriginal and Torres Strait Islander children compared with non-Aboriginal and Torres Strait Islander children (6). Children affected by overweight and obesity are more likely to carry excess weight into adulthood, predisposing them to earlier chronic disease (7).

There is a need to ensure timely program provision to intervene early and promote lifelong healthy messaging. Currently in Australia, healthcare services are not providing adequate multidisciplinary healthy lifestyle programs for children and young people affected by obesity in terms of coverage and place-based considerations. A 2021 study identified 17 services across Australia with only two in rural and remote settings (8). Childhood obesity is a key priority area for policy in many jurisdictions. In response to rising health costs due to obesity-related chronic disease, the WA Department of Health's Sustainable Health Review in 2019 prioritised halting the rise of obesity by July 2024 (9). The Australian National Obesity Strategy 2022–2032 set a target to “reduce overweight and obesity in children and adolescents aged 2–17 years by at least 5% by 2030, [(10), p. 7]” with implementation of supportive healthcare and early intervention to be guided by equity, using genuine partnerships to develop co-designed responses to care (10). To assist with clarity in relation to the impact of comorbidity for those affected by obesity, the Lancet Commission on the definition and diagnostic criteria of clinical obesity has moved away from a dependency on just body mass index (BMI) to define obesity status (11). The consensus definition now redefines obesity as either *pre-clinical obesity*, a condition of excess adiposity without current organ dysfunction or limitations in activities of daily living, or *clinical obesity*, a chronic, systemic disease state directly caused by excess adiposity (11). It is hoped that this clarity will address the unequal access to care and highlight the need for services for those severely affected by excess weight (11).

Intervening in childhood to prevent weight-related adverse health outcomes and to maximise wellbeing into adulthood is critical for children and supported by international recommendations (12, 13). In 2016, the World Health Organization's Report of the Commission for Ending Childhood Obesity recommended providing family-based, multi-component lifestyle weight management services for children and young people affected by overweight and obesity (14). Further, the 2017 United States Preventative Taskforce recommended in its systematic review that all children >6 years old should have access to comprehensive intensive behavioural interventions to achieve improvements in weight status (15). Importantly, this review stated that 26 h of minimum contact time in any program were required to achieve clinically meaningful reductions in weight status (16). The American Academy of Pediatrics Clinical Practice Guideline for the Evaluation and Treatment of Children and Adolescents with Obesity recommends intensive health behaviour and lifestyle treatment from 6 years of age (13).

Creating a multidisciplinary team that focuses on the delivery of strengths-based health-promoting messaging free from judgement and stigma (rather than focusing on body weight and dieting) and that engages the whole family results in optimal outcomes leading to sustained intervention effect (13, 14). Family involvement in such programs is critical, as family-based interventions not only address the inter-generational aspects of obesity but appears to be more cost-effective than parent- and child-only interventions (17). When considering multidisciplinary healthy lifestyle programs in Australia, there remains a lack of equity-focused programs or programs that address accessibility issues by providing care closer to home. Further, even programs shown to be effective have not been sustained or scaled, due to the complexities associated with scaling up interventions (18). It is critical that any program development acknowledges the societal determinants of health and the impacts of colonisation on health outcomes (19). It is also important to understand potential barriers to engagement in healthy lifestyle programs. Past in-depth interviews with program participants from an Aotearoa/New Zealand (henceforth referred to as NZ) study highlight that racism, societal stigma relating to weight, and previous negative experiences of healthcare interactions with weight have often impacted families’ motivation to seek support to make healthy lifestyle changes (20, 21). Conversely, healthcare delivered in a respectful, compassionate manner is a facilitator to engagement with such programs (20). Past studies show that attendance at family-based group sessions is key to positive outcomes, and outcomes are dependent upon culturally safe healthcare provision free from judgement and stigma (20–22).

To address the issues noted and the identified “know-do gap” (23) [also known as the “research-to practice gap” (24)] between extensive evidence and actual service delivery (25), the field of implementation science comprehensively explores the contextual factors and challenges of effectively implementing and then scaling programs. Implementation science is defined “as the scientific study of methods to promote the systematic uptake of research findings and other evidence-based practices into routine practice, and, hence, to improve the quality and effectiveness of health services and care” [(26), p. 1]. This protocol is grounded on well-established implementation science frameworks and methodologies to inform the development and deployment of a healthy lifestyle program for children, young people and their families residing in WA, aiming to address the shortcomings of past and existing programs as outlined previously. “Scale-out” will be the focus of this program, defined as use of strategies to implement, evaluate, improve, and sustain evidence-based interventions delivered in innovative circumstances but distinct to previous interventions (27). The program also considers potential “scale-up” (the evidence-based intervention designed for one setting is expanded to another similar setting, pursuing the same population) (27) should the program demonstrate appropriateness, acceptability, and feasibility.

The proposed healthy lifestyle program is a partnership between Child and Adolescent Community Health, Curtin University, and The Kids Research Institute Australia in Perth, WA. The terms “healthy lifestyle program” and “program” will be used interchangeably. The current project is informed by efficacy studies of a NZ-based healthy lifestyle program called Whānau Pakari, which means healthy, self-assured whānau (wider family unit) (28). This multidisciplinary program was co-created with community in the Taranaki region (a mixed urban–rural region of NZ) and has been evaluated using multiple methods, including a randomised controlled trial (RCT) (29). It focuses on supporting Māori and groups affected by socioeconomic deprivation, given their over-representation in obesity statistics within a public health service provider offering free healthcare for all who are referred (30). The unique aspect of this multidisciplinary program is that it embeds one holistic medical, dietary, physical activity, and wellbeing assessment into the community-based program and offers this assessment in the home or suitable community venue to ensure access to the service, thereby addressing equity. The RCT carried out on program effectiveness demonstrated improvements in weight status at 12 months (greater and for longer duration in those who attended ≥70% of weekly group sessions) (29), cardiovascular fitness in the higher intensity intervention group (22, 29), water intake, and quality of life (22, 29), alongside program cost-effectiveness (31). The RCT also showed high acceptability and high recruitment of target population groups, namely, Māori and those from socioeconomic deprivation (32). Whānau Pakari is now the standard of care for supporting children and young people living with obesity in Taranaki, NZ.

The current project builds on existing knowledge to support and accelerate translation of a version of Whānau Pakari, through partnerships with key healthcare providers and consumer organisations in WA. It aims to adapt and create a culturally secure healthy lifestyle program for the Perth metropolitan context, prioritising Aboriginal and Torres Strait Islander families and those from socioeconomic deprivation during recruitment, utilising a multiple-methods approach. The proposed approach combines a weight-related health assessment and weekly group sessions for 26 weeks (6 months) into one community-based healthy lifestyle program in partnership with Aboriginal and Torres Strait Islander leaders and consumers. The program will build trust through the first “golden appointment” or interaction and ensure screening for weight-related comorbidities to allow for child health outcomes to be optimised, while ensuring a respectful and welcoming environment (33).

The overall research aims are to determine appropriateness, feasibility, and acceptability of the adapted, Perth-based healthy lifestyle program for children and young people and develop the program with key partners for potential scale. Within-subject clinical effectiveness will be captured; however, the key focus is on program effectiveness as a whole.

## 2 Methods and analysis

### 2.1 Study setting

The research will take place in Perth, WA, the fourth most populous city in Australia, with a population of 2.3 million (34). Embedded within Child and Adolescent Community Health (the Metropolitan-wide health service for children and adolescents in Perth), the program has funding from the broader health service provider, Child and Adolescent Health Service, for a 22-month duration. Support for the program is also provided by consumers, research organisations, and Population and Preventive Health of the WA Department of Health to optimise a pathway for sustainment. The project will be delivered within the East Metropolitan Health Service catchment boundary, which has a population where 2.7% (*n* = 21,500) identify as Aboriginal (35). This program aims to complement existing activity of the tertiary hospital-based Perth Children's Hospital Healthy Weight Service (currently experiencing high demand and extensive waitlist) and the Better Health program, a 10-week family-based lifestyle intervention program modified from the UK Mind, Exercise, Nutrition, Do it trial (36). While the focus of this study will be in Perth, it will inform potential scale-up of the program across the metropolitan catchments and WA more broadly.

### 2.2 Study overview

A hybrid type II effectiveness-implementation design will be utilised, using multiple methods and established implementation science frameworks and tools (37). This research will evaluate the feasibility of the evidence-based healthy lifestyle program and undertake the required health system development and program adaptation, resourcing, and capability growth, with the ultimate objective of scale-up with adaptations to address cultural and place-based considerations. Expert involvement is key to the program, which has been reviewed with multiple partners in the community and healthcare system. Advisory and working groups will also be established to work in partnership throughout the program to ensure identified priorities of the end users are addressed. All groups will oversee the program and be involved in specific research studies. Effectiveness of the approach has been demonstrated with the RCT of the NZ-based program (29). It has been noted that legitimately borrowing strength from previous studies with empirical evidence can accelerate and expand the benefit to populations, removing costs and delays of implementation (27). This research will monitor the program changes in line with the NZ evidence base, and core critical elements of the NZ program will be maintained (32). Implementation strategies are noted to be essential when developing an intervention (38) and defined “as methods or techniques developed to facilitate adherence to, and adoption and sustainability of an intervention” [(39), p. 2]. The Replicating Effective Program (REP) framework has been utilised to outline the stages of program evolution by developing a plan for implementing this evidence-based intervention within community, based on the NZ program, early program work, and proposed strategies (Table 1) (40). Table 1 outlines the REP framework adapted to the healthy lifestyle program. Multiple perspectives incorporated in the research studies, including Aboriginal and Torres Strait Islander advisors, children, young people and caregivers, key partners’, and key research and staff members will aid in considering further implementation factors during the program (39). “Key partners” are defined as a range of individuals and organisations within the community and healthcare system, including health staff/management, community support personnel, policymakers, and referrers to the healthy lifestyle program, with various levels of involvement with the program. The term “caregiver” refers to biological, adoptive, foster, single, same-sex, kinship, or legal guardian. Equitable access to health services and identifying and mitigating barriers to engagement for Aboriginal and Torres Strait Islander Peoples are key commitments of this program. This program of work is informed by the WA Aboriginal Health and Wellbeing Framework (2015–2030) (41) and abides by the guiding principles of partnership and access, and equality in health services. The program has been informed by all of the strategic directions set out in the Framework, which centre around culture, namely, prevention and early intervention, promote good health across the life course, a culturally respectful and non-discriminatory health system, individual, family, and community health and wellbeing; a strong skilled and growing Aboriginal health workforce; and equitable and timely access to the best quality and safe care [(41), p.9]. Acknowledging that there is likely to be a broad diversity of representation within the program participants, the research and clinical groups will work to honour the WA Health Equity Impact Statement and Declaration Policy (42) to reduce health inequity in priority population groups referred to the program.

**Table 1:** REP framework adapted to the healthy lifestyle program.

Phase	Activity	Process
Preconditions	Identify need	By 2050, 50% of children and young people are estimated to be affected by overweight or obesity (44). Recommendation 2a of the Sustainable Health Review—“Halt the rise in obesity in WA by July 2024 and have the highest percentage of population with a healthy weight of all states in Australia by July 2029” [(9), p. 48]. A 5% reduction in the prevalence of overweight and obesity has been estimated as leading to cost savings of AUD 7.44 billion in Australia (45–48). National Obesity Strategy states that one in four children are affected by overweight or obesity in Australia (10). Overweight and obesity impact negatively on quality of life during childhood (49). Lack of consumer and equity-focused multidisciplinary programs in WA including programs that address accessibility issues. Current services have long waitlists with restrictions applied or are not resourced to ensure cultural considerations. Identify at risk group: known over-representation in obesity statistics for those from socioeconomic deprivation, Aboriginal and Torres Strait Islander children and young people, and some culturally and linguistically diverse population groups (50), leading to inequity in child health and quality of life outcomes. Community engagement and meetings over 2.5 years to understand local needs.
	Identify effective intervention	NZ-based program: at a participant-level within an RCT, findings demonstrated improvements in weight status at 12 months (29), cardiovascular fitness (22, 29), water intake, and quality of life (22, 29), and from a service and implementation perspective, program cost-effectiveness and increased efficiencies were achieved (31). This approach resulted in greater engagement from Māori and from families affected by socioeconomic deprivation when compared with previous programs (29). Key partners and referrer satisfaction was high, and resulted in greater referrals and reach when compared with other regions of NZ not offering the intervention (32). The program has been implemented as the standard of care in the Taranaki region (a mixed urban–rural region of NZ) for addressing childhood obesity, after demonstrating efficacy on multiple outcome measures. Site visits to the NZ-based program to review suitability in Perth context with delegates from Aboriginal health within health service provider and Aboriginal medical service. Decision made to adapt the NZ-based equity-focused program.
	Identify barriers	Assessment of potential barriers using ERIC (Table 2) to assist in mitigating barriers and enhancing identified enablers to achieve the desired implementation outcomes (51).
	Draft package	Development of the protocol including program and supporting research studies.
Pre-implementation	Community working group	All expert groups will provide input in developing the Perth-based program. Implementation Science group: researchers guiding on program implementation. Cultural Advisory Group: Aboriginal and Torres Strait Islander Elders guiding cultural and place-based considerations of the program. Consumer Advisory Group: caregivers of children and young people above a healthy weight guiding on lived experience aspects. Health Consumers’ Council: support with consumer and community involvement. Clinical Governance Group: subspecialty team guiding best-practice clinical guidelines for weight-related comorbidities.
	Pilot test package	Revising the study protocol. Standard operating procedures developed for program team; staff training curriculum developed. Promotional materials developed—referral form, brochures.
	Orientation	New program staff recruited, onboarded, and trained.
Implementation	Training	Training from the NZ-based Healthy Lifestyle Coordinator and paediatrician with subspecialty expertise.
	Technical assistance	Biostatistical, health economics, database developers.
	Evaluation	Covered in various research studies (Table 3).
	Ongoing support	Through various partnerships with Health Consumers' Council and expert involvement groups.
	Feedback and refinement	Ongoing meetings with expert advisors to refine program and study outcomes.
Maintenance and evolution	Organisation, financial changes	Reviewing further funding opportunities to continue the program. Business case for pilot of contemporary model of care.
	National dissemination	Publishing research findings through peer-reviewed journals and policy briefings.
	Re-customise delivery as need arises	Research findings to guide program evolution.

Table 2 summarises the pre-implementation activities, mapped onto the Expert Recommendations for Implementing Change (ERIC) framework of implementation strategies in preparation for program rollout within local health service provider jurisdictions (43).

**Table 2:** ERIC pre-implementation strategies for healthy lifestyle program development.

Strategy	Activities in pre-implementation phase of the healthy lifestyle program
Access new funding	Clinical service funding approved: Child and Adolescent Health Service for pilot (22 months). Research funding attained (Research Fellow and PhD scholarships), Curtin University, and Western Australian Future Health Research and Innovation Fund and Curtin University Clinician Researcher Training scholarship.
Build a coalition	Leadership and partnership with researchers, consumer advocates, clinician researchers, Aboriginal and Torres Strait Islander Elders, subspecialists and health practitioners within the team, representing numerous sectors within the health system. Situated within Child and Adolescent Community Health, Child and Adolescent Health Service, in partnership with East Metropolitan Health Service.
Change service sites	Healthcare outside hospital walls and into community in the East Metropolitan Health Service geographical catchment. Clinical team site identified. Assessments to occur at home, school sites in partnership with Child and Parent Centres or at Aboriginal Health Team sites. Group sessions to be held at identified school venues.
Conduct educational meetings	Presentations to consumer groups’ different health service providers, researchers within Curtin University, conferences throughout program development phase.
Conduct educational outreach visits	Meetings with schools, Aboriginal medical services, child health nursing, East Metropolitan Aboriginal health services, subspecialists and general paediatricians at tertiary and secondary hospitals, engagement with general practitioners.
Create new clinical team	New clinical positions created, organisational structure created, staff training package created, onboarding new staff to organisation, healthy lifestyle coordinator from NZ trained team in Perth.
Develop academic partnerships	Partnership between Child and Adolescent Community Health, Curtin University, and The Kids Research Institute Australia in Perth, WA. Transdisciplinary expertise recruited in biostatistics, behaviour change, child health, child health policy and advocacy, consumer and community involvement, dietetics, digital health, endocrinology, exercise physiology, health economics, health psychology, health systems change, paediatrics, program evaluation and implementation, implementation science, nursing, nutrition, physical activity, qualitative, quantitative, and multiple-methods research, health equity, and strengths-based community healthy lifestyle program delivery.
Develop and implement tools for quality monitoring	Consult regarding clinical algorithms, embedding in a data management system for client use. Create a client referral form, establish a dedicated website for program information for referrers and families, liaise with communications and policy teams, order key medical equipment, and order physical activity and dietary equipment. Create client tracker, client flow, letter templates, multidisciplinary team templates, referral letters, investigation procedures (e.g., oximetry), and training for staff in investigation. Develop referrer training package. Recruit research team and PhD candidates.
Inform local opinion leaders	Regular meetings with health service providers and Australasian network of health professionals.
Involve executive boards	Meetings and interim reports to the Executive team. Regular program updates at executive meetings.
Remind clinicians	Quarterly newsletters to all potential referrers and regular program updates to Child and Adolescent Community Health staff.
Use an implementation advisor	Local, national, and international implementation advisors recruited.
Visit other sites	Delegates from Aboriginal medical service, Child and Adolescent Community Health including the Aboriginal Health Team visited the NZ program in NZ to determine the appropriateness for a WA setting.

Table 3 provides an overview of studies included in this implementation research project, including implementation strategies and tools being applied.

**Table 3:** Summary of studies evaluating implementation and program participant-level outcomes of the healthy lifestyle program.

Study	(1) Develop the adapted healthy lifestyle program with key partners and Aboriginal and Torres Strait Islander advisors for scale-out		(2) Assess acceptability, appropriateness, feasibility, and local clinical outcomes of adapted healthy lifestyle program pilot			(3) Assess program scalability post-pilot
Aim	Identify critical elements and outcomes to determine program success	Identify cultural and place-based considerations for program success	Evaluate the healthy lifestyle program based on participant outcome measures	Explore program participant experience including program access and appropriateness	Explore program feasibility and scalability from a health system perspective	Create a program package to assist practitioners and policymakers with program scale-up
Study design	Qualitative workshop and qualitative proformas pre-implementation	Qualitative workshop pre-implementation	Quantitative outcomes at baseline, 6 and 12 months. Single-arm, within-subject (primary and secondary) outcomes study^a^	Qualitative focus groups and proformas	Qualitative workshop and qualitative proformas at the end of the program	Qualitative common elements of successfully scaled interventions and interviews with program staff
Participants	*Key partners* Workshop ∼30 participants. Qualitative proforma ∼80 respondents	*Aboriginal and Torres Strait Islander Elders/advisors* Workshop ∼30 participants	*Program participants* Children and young people aged 4–16 years. Estimated *n* = 245 required over 22 months, accounting for 30% drop-out	*Program participants* ∼3 focus groups, 8–12 weeks post-program commencement. ∼3 focus groups, 6 months post-program commencement. Evaluation survey to all participants	*Key partners* Workshop ∼30 participants. Qualitative proforma ∼80 respondents	*Healthy lifestyle program delivery team*. Interviews ∼10 key research and program staff members
Analysis	Barrier and enabler approach of implementation concern using Framework Analysis incorporating the CFIR (53–55)	Barrier and enabler approach of implementation concern using Framework Analysis incorporating the CFIR (53–55)	Mixed model regression to assess within-subject change over time	Barrier and enabler approach of implementation concern using Framework Analysis incorporating the CFIR (53–55). Descriptive statistics—proforma	Barrier and enabler approach of implementation concern using Framework Analysis incorporating the CFIR (53–55)	Framework Analysis (56) incorporating ERIC (43)
Expert involvement	Implementation science research team members	Cultural Advisory Group. Health Consumers’ Council	Clinical Governance Group	Consumer Advisory Group. Health Consumers’ Council	Implementation Science Research Team members	Implementation Science Research Team members. Research team with subject matter expertise
Implementation output	IRLM, RTT	IRLM	—	IRLM (∼8–12 weeks)	ISAT	Program packaging

CFIR, Consolidated Framework of Implementation Research (57); ERIC, Expert Recommendations for Implementing Change (43); IRLM, implementation research logic model (58); ISAT, intervention scalability assessment tool (59); RTT, Readiness Thinking Tool (60).

^a^ [RCT previously conducted within the NZ-based program (29)].

### 2.3 Informed consent, confidentiality, and data management

All participants will provide consent to participate in the relevant studies. A Participant Information and Consent Form (PICF) relevant to each study will be provided to potential participants prior to taking part in the research studies to which they are invited. Depending on the study, the PICF may be provided in person or via email/post. Written informed consent/assent will be obtained on the day for the healthy lifestyle program and in-person workshops, focus groups, and interviews. For online workshops/focus groups/interviews, informed consent/assent will be obtained prior to the session via online forms. Informed consent will be obtained from the participant if age-appropriate or, when the participants are children, from a caregiver. It will be optional for children and young people to sign the consent form (if age-appropriate). For participants taking part in the online qualitative proformas and evaluation survey, the PICF will be at the start for participants to read and select a box that they consent to participate. All potential participants will be able to ask questions at the time of recruitment and/or contact the research team prior to the study with any queries. There will also be an opportunity for further clarification for those participating in the program, workshops, focus groups, and interviews on the day. All participants can withdraw from the studies at any time. Data will be removed at the time of withdrawal if the withdrawal is prior to participating in the workshop/focus groups and analysis.

All qualitative data will be anonymised, and data from the healthy lifestyle program will be de-identified prior to analysis. Study documents will be stored electronically using password-protected files that only investigators and researchers associated with this project have access to. Consent forms and participant data will be stored separately. Records will be stored for a period of 7 years after completion of the research or until the youngest participant turns 25 years of age (52). Once decided that the data are no longer required, they will be destroyed. Confidential and identifying information about participants will not be included in any publications or reports emanating from the project.

### 2.4 Study 1: develop the adapted healthy lifestyle program with key partners and Aboriginal and Torres Strait Islander advisors for scale-out

To identify critical elements and outcomes that will determine program success, especially in relation to place-based and cultural considerations, the following studies will be undertaken.

#### 2.4.1 Identify critical elements and outcomes to determine program success

##### 2.4.1.1 Overview

Before program commencement, important elements and program outcomes will be identified to provide clear measures of program success with key partners.

##### 2.4.1.2 Aim

Identify critical elements and outcomes to determine program success.

##### 2.4.1.3 Study design

A qualitative research design including a 3 h workshop and qualitative proformas [open-ended (free-text) surveys] (53–55) during the pre-implementation phase.

##### 2.4.1.4 Participants

The workshop will include approximately 30 participants and approximately 80 participants will be invited to take part in the qualitative proformas. For the purposes of this study, key partners for the workshop are defined as participants in health organisation leadership roles and healthcare professionals. The qualitative proformas will incorporate a wider group of participants covering our main definition of key partners outlined earlier.

##### 2.4.1.5 Recruitment

Participants will be purposively recruited through Child and Adolescent Community Health, and other key groups, alongside potential referrers. Key partners will be invited to participate in the workshop and online qualitative proforma via email. The online qualitative proforma invites will not include the workshop participants. Background information will be provided regarding the workshop for the online proforma. Weekly follow-up reminder emails to complete the proforma will be made for 4 weeks until it closes. Study data for the proforma will be collected and managed using the Research Electronic Data Capture (REDCap, Vanderbilt, National Institutes of Health) tools hosted at WA Department of Health (61, 62). REDCap is a secure, web-based software platform designed to support data capture for research studies, providing (1) an intuitive interface for validated data capture, (2) audit trails for tracking data manipulation and export procedures, (3) automated export procedures for seamless data downloads to common statistical packages, and (4) procedures for data integration and interoperability with external sources (63).

##### 2.4.1.6 Workshop format

Members of our implementation science team will facilitate the workshop (pre-implementation). The workshop will aim to understand program outcomes and how the outcome measures of success would be weighted by importance. The workshop will be supported by digital audience interaction (Slido, Webex by Cisco, London, 2024) to promote active participation and will allow confidential answers to be made. Slido is an audience engagement tool used in real-time meetings, used for ranking, word clouds, and open text (64). The workshop will be audio-recorded.

After introductions and an outline of aims, the workshop will include a presentation about the program and include potential program sustainment and scalability. The organisational Readiness Thinking Tool on implementing the program will be administered to participants (60). Following this, the group will be split into smaller groups to identify critical program success factors and outcome measures to feed back to the group. The original group will re-form to review each group's rankings to reach a consensus on these measures.

##### 2.4.1.7 Qualitative proforma format

Qualitative proformas enable individuals to provide detailed information (53–55) and are an established rapid approach (65). The measures of success from the workshop will be provided to the wider key partner group.

##### 2.4.1.8 Analysis

Identified outcomes for program success will be categorised as participant, service, and program implementation outcomes (66). These measurements will inform the feasibility of the program alongside more in-depth economic measures. Multi-modal data (e.g., recordings, butchers’ paper, proformas, Slido) will be analysed based on Smith et al. (53) barrier and enabler approach to identify implementation concern that combines Framework Analysis (56) and the Consolidated Framework of Implementation Research (CFIR) (57). This is a flexible approach to qualitative data analysis and has been adapted to suit different contexts, as evidenced by Smith et al. (54). Framework analysis is suitable as it was developed to address specific questions and has an applied research approach that can inform both policy and practice (56). The CFIR is a recognised framework for assessing challenges and drivers in the process of implementing interventions and can be applied to any stage of the evaluation process (54). The analytical process will follow five stages: familiarisation, identifying a thematic framework [the CFIR will be used to identify barriers/enablers of implementation concern (53–55)], indexing, charting, and mapping and interpretation. NVivo 14 will be used to manage the data (67). Permission has been provided by Lumivero to use/mention NVivo as a software tool with all the related studies (67).

#### 2.4.2 Identify cultural and place-based considerations for program success

##### 2.4.2.1 Overview

When considering scale-out of an evidence-based program, it is important that cultural and place-based considerations are taken into account in any adaptation (68).

##### 2.4.2.2 Aim

Identify cultural and place-based considerations for program success.

##### 2.4.2.3 Study design

A qualitative workshop.

##### 2.4.2.4 Participants

Inclusion criteria: Aboriginal and Torres Strait Islander advisors (*n* ∼ 30) interested in the cultural safety of the program.

##### 2.4.2.5 Recruitment

Participants will be purposively recruited through the program's collaborations. Snowball sampling will be welcomed from initial contacts, ensuring diverse participant perspectives.

##### 2.4.2.6 Workshop format

An Aboriginal engagement facilitator will conduct the 3-h workshop with support from research team members (pre-implementation). The workshop will commence with an introduction and setting of participation “guidelines” and then review barriers and mitigation strategies for cultural aspects. The format will be a mixture of participants responding as a whole group and working in smaller groups. The workshop will be audio-recorded if consent is obtained, with Butcher's paper for groupwork note-taking.

##### 2.4.2.7 Workshop schedule

Areas to be covered: expectations of the program; considerations for developing a healthy lifestyle program for the Perth metropolitan context and how the model can best meet the needs of the community; potential barriers and enablers to engaging with the program; critical elements of the program that will ensure cultural safety and appropriateness; and specific questions around mitigation strategies, group sessions, and planning. Participants will be reimbursed in line with consumer payment guidelines (69) and cost of transport.

##### 2.4.2.8 Analysis

The workshop will be analysed using a barrier and enabler approach, combining Framework Analysis and the CFIR (53–55) (see Section 2.4.1.8 Analysis).

### 2.5 Study 2: assess acceptability, appropriateness, feasibility, and local clinical outcomes of adapted healthy lifestyle program pilot

To determine the acceptability, appropriateness, and feasibility of the healthy lifestyle program, the following studies will be undertaken.

#### 2.5.1 Evaluate the healthy lifestyle program based on participant outcome measures

##### 2.5.1.1 Overview

The multidisciplinary healthy lifestyle program will be conducted as a 12-month pilot and includes home-based weight-related assessments and weekly group activity sessions.

##### 2.5.1.2 Aim

Evaluate the healthy lifestyle program based on participant outcome measures.

##### 2.5.1.3 Study design

The previously outlined studies will inform program design. This research builds on a decade of efficacy studies including an RCT to determine efficacy within the NZ-based program (28, 29). This program scale-out will be evaluated by a single-arm study, with outcomes determined by within-subject change over time.

##### 2.5.1.4 Participants

Inclusion criteria: Children and young people aged 4–16 years residing in the East Metropolitan Health Service catchment area referred to the program, with a BMI ≥95th percentile (obesity cut-point), or those with ≥85th percentile (overweight cut-point) with weight-related comorbidities. These cut-points are used in Child and Adolescent Community Child Health, based on Centers for Disease Control (CDC) and Prevention (70) and the American Academy of Pediatrics recommendations (13). Exclusion criteria include significant medical or psychological conditions leading to inability to engage with the program; children/young people without a committed family member, or those with significant weight-related health conditions requiring tertiary input.

A total sample size of 245 is required to accommodate for an estimated 30% drop-out rate as seen in the NZ study (required *n* = 171) over 22 months (29). This sample size can show statistical significance for a mean change in BMI standard deviation score (SDS) of 0.1, with a standard deviation of 0.4 (small effect size), power of 90%, and an alpha of 0.05. A meta-analysis of childhood obesity interventions showed that a change of −0.1 BMI SDS can lead to improvements in cardiovascular and metabolic outcomes (71). Sample size was determined using a two-tailed *t*-test with a difference between two dependent means using change from baseline to 12 months.

##### 2.5.1.5 Recruitment

**Figure 1:**
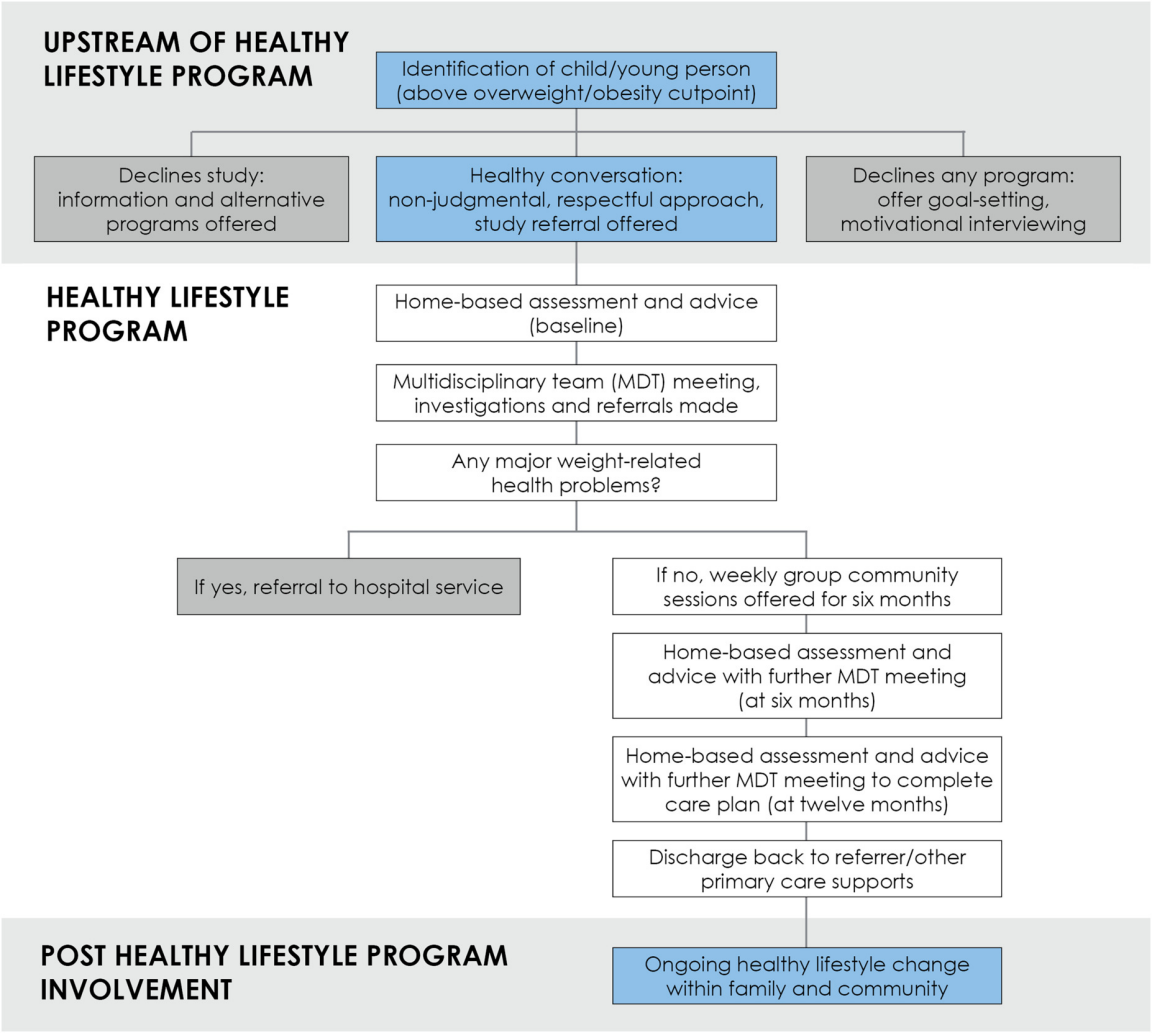
The healthy lifestyle program pathway.

Referrals will come from health professionals within the identified communities, including child and school health nurses, Aboriginal and Torres Strait Islander community-controlled health services, and other professionals involved in the child or young person's care. It is anticipated that some referrals will come from the School Entry Health Assessment at ages 4–5 years old, where growth screening is undertaken. This will provide opportunities to intervene early, which is important given that long-term outcomes are more favourable in younger children (<10 years) (22). Self-referrals will also be received. Promotion of the program will be through Child and Adolescent Community Health, key relevant partners, and primary care organisations and schools. Pathway components are shown in Figure 1.

##### 2.5.1.6 Program format

The program will undertake weight-related assessments including selected outcome measures at baseline, 6 and 12 months (implementation). Weekly group sessions for participants will be delivered for 6 months. All children referred will be seen in the family home or community location by the Healthy Lifestyle Coordinator (a health professional trained in focused weight-related assessment, supported by a paediatrician). Removal of a hospital-based appointment with a specialist will enable access to the service for larger numbers and priority groups and “demedicalise” the process. An Aboriginal health practitioner (employed by the program) will support families where indicated and needed. The team comprises dietitians, an exercise physiologist, and a psychologist, with paediatrician oversight. Cultural safety of the program will be ensured with Aboriginal and Torres Strait Islander guidance and leadership throughout the project. Table 4 outlines the support provided, with a similar format to the NZ program (28).

**Table 4:** Support to be provided for participants during the healthy lifestyle program.

Support to be provided during the program
Baseline, 6- and 12-month assessments with healthy lifestyle change advice in the home or a community centre (blood tests only when indicated)
Questionnaire review (team), multidisciplinary team meeting following each assessment—review and action of alerts
Community visit/telehealth/phone contact within first month from exercise physiologist and/or dietitian if indicated and accepted, ±Aboriginal and Torres Strait Islander health practitioner (±ongoing support)
Exercise physiologist/dietitian review of progress at 6 months (seen at group if attending)
±Keyworker engagement depending on progress at 6 months
Weekly activity and education sessions for 6 months (total of 26 h contact time)
Total home/community visits offered over 1 year = 3 minimum

##### 2.5.1.7 Assessment format

The children and young people will be offered a healthy lifestyle check by a healthy lifestyle coordinator at baseline and 6-monthly for 12 months. Table 5 outlines the key physical assessment measures. Of note, weight status will not be an emphasis with participants.

**Table 5:** Key elements of the physical assessment measures during the program evaluation.

Key assessments	Baseline	6 months	12 months
Resting heart rate	✓	✓	✓
Blood pressure (using Welch Allyn portable sphygmomanometer with flexiport reusable BP cuffs of appropriate size)	✓	✓	✓
Height (to 0.1 cm using average of three readings on Seca 213 portable stadiometer)	✓	✓	✓
Weight (to 0.1 kg using Seca 813 digital scales)	✓	✓	✓
Acanthosis nigricans screen	✓	✓	✓
Ear, nose, and throat examination (using Welch Allyn portable otoscope)	✓	✓	✓
Self-report of Tanner pubertal stage (or from parent in very young children)	✓	✓	✓
Accompanying adult's height and weight	✓		✓
Blood sampling^a^ [only where indicated, may include liver function tests, glycated haemoglobin (HbA1c), and lipid studies]	±	±	±

^a^ Blood sampling will be undertaken only as part of best-practice clinical care (see NZ program methodology paper) (28).

Information will be gathered regarding ethnicity, past medical history, medical conditions, and family history. Questionnaires that will be administered (at baseline, 6, and 12 months) are outlined in Table 6.

**Table 6:** Standardised questionnaires in the healthy lifestyle program evaluation.

Questionnaire	Description
Paediatric Quality of Life Inventory (PedsQL)™ (77–81)	Designed to evaluate health-related quality of life in children/adolescents—has been extensively validated. Self-report and/or caregiver proxy report, depending on age of child or young person (Supplementary Table S1)
Strengths and Difficulties Questionnaire (SDQ) (82)	Behavioural screening questionnaire. Self-report and/or caregiver proxy report, depending on the age of the child or young person (Supplementary Table S1)
CHU-9D (83, 84) and CHU-9D proxy (85) and under 5's (86)	Instrument and proxy version (for caregivers to complete for younger children) utilised to obtain quality-adjusted life years directly for use in cost-utility analysis. (Supplementary Table S1)
Physical activity questionnaire	Questionnaire developed using format similar to aspects of Children's Physical Activity Questionnaire (87)
Physical activity and nutrition knowledge	Open-ended qualitative survey questions [adapted from the NZ Nutrition survey (88)]
Health-seeking behaviour	Closed questions regarding presentations to primary care and hospital in the past 6 months for child/young person similar to resources utilisation questions (89)
Sleep-Related Breathing Disorder Scale from Pediatric Sleep Questionnaire (PSQ-SRBD) (90)	Validated sleep questionnaire for sleep-disordered breathing
Multi-pass 24 h food recall (91)	Dietary questionnaire assessing food and beverage intake in the preceding 24 h
Two-item food insecurity scale (92)	Brief screening tool for food insecurity
ScreenQ (93)	To determine the quantity and quality of screen time

REDCap will be utilised for data collection. Data will be reviewed monthly for validity and completeness. The questionnaire will include alerts for data outside acceptable medical parameters (e.g., elevated blood pressure percentiles) for discussion at multidisciplinary team meetings to individualise patient recommendations. Progress will be captured on a multidisciplinary team worksheet and results of each assessment will be reviewed and further referrals made, as required.

##### 2.5.1.8 Calculations

Height and weight percentile, BMI, BMI percentile, BMI SDS, and BMI percentage of the 95th percentile will be calculated using the CDC learning management system values. These are based on sex-specific normative data from the 2000 CDC growth charts, or the 2022 CDC extended BMI-for-age growth charts for BMI over the 95th percentile (13, 72). Blood pressure percentiles will be calculated based on the National Institutes of Health's Fourth Report on the Diagnosis, Evaluation, and Treatment of High Blood Pressure in Children and Adolescents (73), with management as determined in Hypertension Canada's 2017 Guidelines for the Diagnosis, Assessment, Prevention, and Treatment of Pediatric Hypertension (74). Level of household disadvantage will be calculated based on Socio-economic Indexes for Areas (75).

##### 2.5.1.9 Analysis

Demographics and important clinical features will be compared between participants remaining in the study to those who declined further engagement prior to follow-up. Depending on the distribution of the data, independent *t*-tests, the Mann–Whitney *U* test, chi squared test, or Fisher's exact test will be used for statistical comparisons between these two groups. Patterns of missing data will be explored, and when appropriate, multiple imputations will be used to handle missing predictor or confounding variables (assuming data are missing at random) (76). Missing data for outcomes will not be imputed but will be handled using a mixed effect model approach that allows for within-subject missing data. A linear mixed model regression will be used to assess change at each time point for continuous normative data, with participant entered as a random effect to account for within-subject repeated measures. A generalised linear mixed model will be used for outcomes with non-normal distributions such as skewed, binary, categorical, ordinal, and count data, with the link function and family determined by the data distribution and model fit. Models will be adjusted for age, gender, socioeconomic status, and ethnicity. Subgroup analyses are planned for age-related dietary patterns, physical activity, and wellbeing measures (including screens and sleep). Baseline characteristics will be summarised using descriptive statistics. Continuous variables will be described as numbers of observed and missing values, mean, standard deviation, median, minimum, and maximum. Categorical variables will be described as frequencies and percentages.

##### 2.5.1.10 Outcomes

Primary outcome measures are change in BMI SDS at 6 and 12 months post-enrolment and change in BMI percentage of the 95th percentile (94). Secondary outcomes include changes in health-related quality of life, dietary intake, physical activity, screen use, knowledge of the benefits of physical activity and healthy eating, and blood pressure and resting heart rate at 6 and 12 months. The Child Health Utility 9D (CHU-9D) instrument (83) will obtain quality-adjusted life years for use in cost-utility analysis (95), and additional economic outcome measures will be examined (e.g., health-seeking behaviour and cost of intervention per person). In accordance with the WA Aboriginal Health and Wellbeing Framework, when ensuring equitable services, two key outcomes are (timely) access to services and cultural appropriateness (41). These will be measured through uptake of participants identifying as Aboriginal and Torres Strait Islander and diverse ethnicities and through focus groups with participants (see Section 2.5.2).

#### 2.5.2 Explore program participant experience including program access and appropriateness

##### 2.5.2.1 Overview

To capture participant experience during the program and determine program acceptability alongside identified successes/improvements of program design and delivery, focus groups and an evaluation survey will be conducted.

##### 2.5.2.2 Aim

Explore program participant experience including program access and appropriateness.

##### 2.5.2.3 Study design

A mixed-methods design using focus groups and an evaluation survey.

##### 2.5.2.4 Participants

Focus groups will be conducted at 8–12 weeks and the 6-month time points with up to 50 program participants or family members (to accommodate withdrawals). The number of focus groups will be dependent on group format (e.g., mixture of children, caregivers, and young people). Aboriginal and Torres Strait Islander participants and caregivers will be offered the choice of a group facilitated by an Aboriginal facilitator or to join the broad focus groups. Inclusion criteria: 8–12 weeks—children and young people who have been participants in the program for 8–12 weeks and their caregivers; 6 months—participants in the program who have completed their 6-month assessments. The same participants are not required at both time points. All participants will be invited to take part in the evaluation survey, including those who declined or dropped out.

##### 2.5.2.5 Recruitment

Participants will be recruited through the program. A purposive stratified sample for the focus groups will ensure a balanced representation of participants at both time points. All participants will be invited to complete the survey at the time point at which they leave the program.

##### 2.5.2.6 Focus group format

Focus groups will be offered in-person and online during the implementation of the program. The option for shared and/or separate groups will also be offered for young people. A facilitator from the partner consumer organisation will conduct the focus groups with support from research personnel (to help with obtaining consent and answering study queries). A physical activity facilitator will attend the in-person focus groups to conduct activities with the children and young people, if required. The duration of each focus group will be approximately 1–1.5 h and will be recorded for later analysis. Caregivers will be reimbursed for their focus group participation in line with consumer payment guidelines (69).

##### 2.5.2.7 Focus group schedules

Areas that will be addressed in the 8–12 week and 6 month focus groups include participants’ program experiences, program place-based considerations for Perth metropolitan context, barriers/enablers experienced engaging with a healthy lifestyle program, exploring cultural safety and appropriateness, and program improvements identified as they evolve.

##### 2.5.2.8 Evaluation survey format

The evaluation survey will be adapted with considerations for local use from the NZ study design (96) and hosted on REDCap. It will include quantitative and qualitative questions. Participants will be invited to participate via email, text, or phone.

##### 2.5.2.9 Analysis

The focus groups and the qualitative aspects of the evaluation survey will be analysed using a barrier and enabler approach, combining Framework Analysis and the CFIR (53–55) (see Section 2.4.1.8 Analysis). The quantitative aspects of the survey will be analysed using descriptive statistics.

#### 2.5.3 Explore program feasibility and scalability from a health system perspective

##### 2.5.3.1 Overview

Upon completion of the program, program feasibility and scalability will be assessed. The findings will inform a contemporary model of care evaluative framework for the program and scalability to facilitate embedding the program into usual care if deemed effective, or other jurisdictions.

##### 2.5.3.2 Aim

Explore program feasibility and scalability from a health system perspective.

##### 2.5.3.3 Study design

A qualitative research design including 3 h workshop and qualitative proformas at the end of the program.

##### 2.5.3.4 Participants

The workshop will include approximately 30 participants and approximately 80 participants will be invited to take part in the qualitative proformas. The workshop and proforma participants criteria will follow the same format as outlined per Section 2.4.1.4.

##### 2.5.3.5 Recruitment

Participants will be purposively recruited as per Section 2.4.1.5.

##### 2.5.3.6 Workshop format

Members of our implementation science team will facilitate the post-program workshop. The workshop will include a scalability assessment of the program post-completion. The workshop will be supported by Slido to promote active participation and will allow confidential answers to be made. The workshop will be audio-recorded.

The workshop will start with introductions of facilitators and attendees, aims, and workshop guidelines. The workshop will include a presentation to communicate program outcomes post-program completion. The ISAT (59) will then be applied to the program outcomes and administered to participants to complete. Overarching discussion will be conducted in relation to the outcomes achieved, feasibility of the program, and suitability for scale-up.

##### 2.5.3.7 Qualitative proforma format

The post-program qualitative proformas will follow the same format as per Section 2.4.1.7.

##### 2.5.3.8 Analysis

Multi-modal data (e.g., recordings, butchers’ paper, proformas, Slido) will be analysed using a barrier and enabler approach, combining Framework Analysis and the CFIR (53–55) (see Section 2.4.1.8). The scalability of the program will be assessed in accordance with the ISAT tool and data from the workshop (59).

### 2.6 Study 3: assess program scalability post-pilot

Once the pilot is completed, an analysis of suitability for potential scale-up will be undertaken.

#### 2.6.1 Create a program package to assist practitioners and policymakers with program scale-up

##### 2.6.1.1 Overview

To determine key elements of program operationalisation into a program package, a Common Elements Approach will be used (97). This approach provides a framework for the synthesis and distillation of “common ingredients” of interventions or implementation to enable exploration of their merit (97). The program package will enable the potential embedding of the program into the health system and facilitate scale-up by identifying common elements of program packages and toolkits available for successfully scaled and sustained programs, identifying key aspects of program operationalisation, and proposing tools and resources for inclusion in a healthy lifestyle program package.

##### 2.6.1.2 Aim

Create a program package to assist practitioners and policymakers with potential scale-up.

##### 2.6.1.3 Study design

A qualitative design utilising the Common Elements Approach (97) will enable the identification and review of program packages available for successfully scaled and sustained childhood obesity interventions. Key elements of operationalisation of this program will be identified via in-depth interviews with key program research and clinical and operational staff and then translated and matched to program package elements using ERIC (43, 97, 98).

##### 2.6.1.4 Participants

Up to 10 interviews will be conducted with key staff members including program staff, research team, and health organisation leads.

##### 2.6.1.5 Recruitment

Participants will be recruited purposively from Child and Adolescent Community Health, the program research, and service teams via email.

##### 2.6.1.6 Interview format

Semi-structured interviews will be conducted on timeline and processes for program operationalisation, program adaptations, and elements perceived to be critical to success.

##### 2.6.1.7 Analysis

Common elements of program packages available for successfully scaled interventions will be identified and key elements of program operationalisation interpreted from in-depth interview content. Using Framework Analysis (56), each source will be coded to ERIC (43) in NVivo 14 and matched to suitable tools/resources for inclusion in the program package.

## 3 Discussion

This research aims to improve the quality, research, clinical capabilities and impact of interventions within, and alongside the community in relation to addressing scale-out of healthy lifestyle programs with cultural and place-based considerations. The program in focus is expected to extend the evidence base from the NZ-based Whānau Pakari program and promote the adaptability of this concept to other contexts with novel cultural considerations. The study will be informed by implementation science concepts and frameworks to ensure optimising this work for sustainment and scale. Applying the REP framework embedding an implementation strategy throughout the course of the research will assist in determining key considerations for potential scale-up. Outcomes will be considered from a participant, service, and program implementation outcomes perspective (66). The research has been designed to explore the outcomes of the program and to consider different aspects that may impact the success of the program by prioritising and privileging the voices of end users, Aboriginal and Torres Strait Islander advisors, and key partners as well as research and clinical staff to guide the process. Using a cultural lens is paramount for a program that prioritises Aboriginal and Torres Strait Islander families.

Few evidence-based multidisciplinary programs address scale-up and most are not embedded into standard practice (18). The current study addresses this “know-do gap”, building on a decade of evidence from a community healthy lifestyle program and learning from 3 years of relationship-building with various organisations and community to ensure meaningful partnership in Perth. It also draws on findings from the proposed multiple-methods projects to inform evaluation and implementation. This project has a varied team of investigators to assist with ethical considerations and transfer knowledge across disciplines (99). Further strengths include the team's expertise and the extensive community participation and leadership—consumer involvement has taken place since the conception of the program to listen and learn as to the service needs and gaps from a consumer perspective. Expert involvement will continue throughout the program via a Cultural Advisory Group, a Consumer Advisory Group, a Clinical Governance Group, and the Implementation Science Research Team. The critical success factors of the NZ program have been applied (e.g., community champion, clinical champion, multidisciplinary team) (32). The research team comprises 4 Aboriginal and 17 non-Aboriginal and Torres Strait Islander researchers with complementary skillsets and experience.

Limitations may include response rates to all aspects of the program (e.g., referrals to the program, uptake in the workshops, focus groups, and proformas). Lessons learnt from previous research undertaken with the NZ program on health system and engagement barriers are anticipated (20, 96, 100). The interview study with 64 NZ program participants found that health system factors that affected engagement were national-level policy directives pertaining to the expectations of referrals from health professionals, funding constraints, lack of coordination between services, health system navigation, and the cost of primary care (100). A further interview study with 76 past users of the NZ program found that personal factors that affected engagement were chronic life stressors, negative societal norms of weight and body size resulting in a reluctance to ask for support, and historical negative experiences of healthcare (20). A survey with 71 program participants and caregivers highlighted the convenience of the session location, when other family priorities were considered to be the most important, and families identifying as Māori reported past experiences of healthcare as a key factor influencing their decision to attend (96). Mitigation strategies will be considered to assist these aspects through our various partnerships and through drawing on implementation aspects (e.g., ERIC, Table 2). Similar to the NZ program, this program has also been designed to explore barriers throughout: pre-inception with key partners and Aboriginal advisors, during the program with program consumers, and post-program with key partners and research and program staff. Various modes have also been utilised (e.g., workshops, focus groups, evaluation survey, and interviews) to learn and continuously improve the program. Ongoing partnership with health service providers with regular updates will result in final decisions relating to sustainment and scale. Community engagement will also be considered, including uptake and retention of participants.

Despite the well-known benefits of consumer and community involvement, research has shown that there is a need to address power inequalities in partnership sharing, a lack of diversity and inclusion, and criticisms noted about the true nature of consumer and community involvement in practice (101). Aboriginal participants will be prioritised in the program. The program will recruit a Cultural Advisory Group to address cultural and place-based considerations of the program alongside Aboriginal Governance. A Consumer Advisory Group will also be recruited to represent children, young people, and caregivers with lived experience. The program's research team has experienced researchers with consumer and community involvement expertise who will be supporting the Cultural Advisory Group and Consumer Advisory Group. Partnering with the Health Consumers’ Council, who have a long track record of working with consumers, ensures a neutral stance. The Health Consumers’ Council's Aboriginal facilitator will facilitate the Cultural Advisory Group and Consumer Advisory Group meetings and the focus groups with program participants alongside research team members. The program's commitment to genuine meaningful involvement and partnership aims to ensure that power inequalities are recognised throughout the operationalisation of the program.

This program has the potential to provide a state-wide solution by providing equity-focused healthy lifestyle programs to address child obesity. Given its embedded nature within health services, this program will have immediate translation, reaching hundreds of children and families in the pilot, with the ability to engage with many more across communities, also allowing for increased competency in supporting children living with overweight and obesity. This research has the potential to inform the development of equity-focused contemporary models of care closer to home for children and young people more broadly, at a time when Australasia is facing significant health systems change.

## 4 Conclusion

This research will determine whether a community-based multidisciplinary healthy lifestyle program for children, young people, and their families in Perth, WA, informed by a variety of partners will result in improved health outcomes, especially among Aboriginal and Torres Strait Islander families. Acceptability and feasibility of this program scale-out will be determined by evaluating participant, service, and program implementation outcomes. Utilising implementation science, consumer and community involvement, strong community partnerships, and an understanding of place-based and cultural considerations, this program will inform potential scale-up of equity-focused healthy lifestyle programs more broadly.

## Notes on language use

While the Aboriginal Empowerment Strategy—Western Australia 2021–2029 (102) recommends reference to Aboriginal people, this project refers to Aboriginal and Torres Strait Islander Peoples (103) in recognition of staff and families’ desire to be represented in this work. We acknowledge the use of the term “obesity” is problematic for some within the community and use this term in this context to communicate with a biomedical audience.

## Collective statement

The research team aspires to achieve research that is by-community-for-community. It involves diverse researchers and advisors from transdisciplinary backgrounds, with a commitment to strength-based research that prioritises and privileges child, young people, and family voices alongside the community. The group have a commitment to working towards health equity and working in genuine meaningful partnership.
