# Supplementary material for: Acceptability, feasibility, and program outcomes of an equity-focused, adapted community-based healthy lifestyle program for children, young people, and their families in Perth, Western Australia: an implementation hybrid research protocol
Source: Front Health Serv. 2025 Jul 17;5:1604809. doi: 10.3389/frhs.2025.1604809 (PMC12310583; doi:10.3389/frhs.2025.1604809)
Supplement: Supplementary file 1 [file Table1.pdf]

SUPPLEMENTARY TABLE 1. Standardised questionnaires being used across the age-groups.

Client Age (years)	SDQ: Youth version (self-complete)	SDQ: Caregiver Version proxy (years)		PedsQL: Self Report (years)			PedsQL: Caregiver Report (years)				CHU-9D: Self Report (years)	CHU-9D: Caregiver (proxy) version[a]	
		4-10	11-17	5-7	8-12	13-18	2-4	5-7	8-12	13-18	≥7	Standard proxy	Proxy[b]
**4**		✓					✓+						✓+
**5-6**		✓		✓+				✓+				✓+	
**7**		✓		✓+				✓+			✓+		
**8-10**		✓			✓+				✓+		✓+		
**11-12**	✓		✓		✓+				✓+		✓+		
**13-17**	✓		✓			✓+				✓+	✓+		

[a]If the child or young person is unable to complete any questionnaire (e.g. is non-verbal), the caregiver will complete a proxy version.

[b]With guidance notes for under 5 years.

Key: ✓ = Yes, for all program participants, ✓+ = Yes, if consented to research.
